# Trends in HIV & syphilis prevalence and correlates of HIV infection: results from cross-sectional surveys among women attending ante-natal clinics in Northern Tanzania

**DOI:** 10.1186/1471-2458-10-553

**Published:** 2010-09-13

**Authors:** Yusufu Kumogola, Emma Slaymaker, Basia Zaba, Julius Mngara, Raphael Isingo, John Changalucha, Patrick Mwidunda, Daniel Kimaro, Mark Urassa

**Affiliations:** 1 National Institute for Medical Research, PO Box 1462, Mwanza, Tanzania; 2 Population Studies Department, London School of Hygiene and Tropical Medicine, 49-51 Bedford Square, London, WC1B 3DP, UK; 3 District Health Department for Magu, Magu District Council, Magu, Tanzania; 4 City Health Department, Mwanza City Council, Mwanza, Tanzania

## Abstract

**Background:**

Sentinel surveillance for HIV in ante-natal clinics (ANC) remains the primary method for collecting timely trend data on HIV prevalence in most of sub-Saharan Africa. We describe prevalence of HIV and syphilis infection and trends over time in HIV prevalence among women attending ante-natal clinics (ANC) in Magu district and Mwanza city, part of Mwanza region in Northern Tanzania. HIV prevalence from ANC surveys in 2000 and 2002 was 10.5% and 10.8% respectively. In previous rounds urban residence, residential mobility, the length of time sexually active before marriage, time since marriage and age of the partner were associated with HIV infection.

**Methods:**

A third round of HIV sentinel surveillance was conducted at ante-natal clinics in Mwanza region, Tanzania during 2006. We interviewed women attending 27 ante-natal clinics. In 15 clinics we also anonymously tested women for syphilis and HIV infection and linked these results to the questionnaire data.

**Results:**

HIV prevalence was 7.6% overall in 2006 and 7.4% at the 11 clinics used in previous rounds. Geographical variations in HIV prevalence, apparent in previous rounds, have largely disappeared but syphilis prevalence is still higher in rural clinics. HIV prevalence has declined in urban clinics and is stable in rural clinics. The correlates of HIV infection have changed over time. In this round older age, lower gravidity, remarriage, duration of marriage, sexual activity before marriage, long interval between last birth and pregnancy and child death were all associated with infection.

**Conclusions:**

HIV prevalence trends concur with results from a community-based cohort in the region. Correlates of HIV infection have also changed and more proximate, individual level factors are now more important, in line with the changing epidemiology of infection in this population.

## Background

We describe the prevalence of HIV and syphilis infection and trends over time in HIV prevalence among women attending ante-natal clinics (ANC) in Magu district and Mwanza city, part of Mwanza region in Northern Tanzania.

Sentinel surveillance for HIV in ANC is the primary method for collecting timely trend data on HIV prevalence in most of sub-Saharan Africa [1,2] and has provided useful data to national programmes in Tanzania [3] and elsewhere [4-6]. Estimates of epidemic trends based on the Estimation and Projection Package (EPP) model rely on ANC prevalence data [7]. In the last decade population-based surveys have collected information on HIV status. These can provide more accurate point estimates of HIV prevalence but are more expensive and so conducted less frequently than ANC surveillance. Population-based surveys supply rich background data together with HIV status but the samples are not designed to provide detailed information at the local level.

ANC estimates are known to be biased in the younger age group by selection of sexually active women [8], in older age groups by HIV-related sub-fertility [9] and can be affected by contraceptive prevalence [10,11]. The nature of such biases can change with epidemic stage and in response to behaviour change. Where there are good data on the difference between the general population and ANC attenders simple adjustments can be used to remove differences between the two estimates [12,13].

Comparisons of national data with current ANC estimates have shown that in most countries the recent ANC data tends to overestimate prevalence in the general population [14]. Tanzanian national ANC results have a reasonable correspondance with the population based survey conducted in 2007 [15]. The observed difference is largely because the national sentinel surveillance scheme is not representative of the whole country [15]. There are no surveillance clinics in Mwanza region but the National Institute for Medical Research has conducted various ANC surveys since 1991 [12,16].

Most national HIV surveillance collects only limited background information, such as age and parity. Additional information on the women who give blood for surveillance purposes is valuable for interpreting trends and for assessing changes over time in selection biases at ANC.

Special studies in Malawi [17,18] and Tanzania [12,16,19] have successfully collected extra information from women attending ANC in areas where there are also data available for the general population. Such information was used to identify correlates of HIV infection among pregnant women and to compare the characteristics, and HIV prevalence, among pregnant women and those in the communities around the clinics.

In the Magu and Mwanza ANC study, data have been collected during three rounds of sentinel surveillance at ANC in 2000/1, 2002 and 2006. Results from the first two rounds [16] showed it was possible to characterise women at high risk of HIV infection using simple data on marital status and sexual behaviour collected during a short interview prior to their participation in anonymous unlinked surveillance. HIV prevalence was 10.7% overall and there was no evidence of change between the two rounds. Prevalence was highest in city clinics, lowest in rural clinics and HIV infection was associated with having lived in an urban area at some time in the past. The proportion in the community reporting a recent change of residence was also associated with HIV infection. Individual level factors were associated with infection. Having a partner less than 10 years older was associated with lower odds of infection. Time since first marriage and the time spent sexually active prior to marriage were independently and positively associated with infection. Once these were controlled for, age at first sex was positively associated with infection. Results concurred with those from the local cohort in Kisesa ward [16].

The third round of data collection was designed to provide information on access to and uptake of VCT in ANC, to investigate whether the introduction of VCT may have biased attendance patterns and therefore surveillance estimates, and to update the information from previous rounds on prevalence trends and correlates of infection. In this paper we provide the updated HIV prevalence estimates and trends over time. We have identified correlates of infection in 2006 and compared these to the factors found to be associated with HIV status in the earlier rounds. We compare the HIV prevalence obtained in ANC with that from the Kisesa cohort.

## Methods

In each round, in all participating clinics, women attending ANC were asked to participate in a face to face interview. In the first two rounds, blood was taken for diagnostic syphilis tests and the surplus was used for anonymous HIV tests, these methods are described in detail elsewhere [16].

### 2006 Sample design

An aim of the study, reported elsewhere [20], was to establish whether the availability of HIV testing influenced women's clinic choice, and whether those choices improved access to services to prevent mother to child transmission (PMTCT) in this area and/or biased surveillance estimates. HIV testing was available at 4 ANC in 2006. We surveyed three types of clinic: those offering no diagnostic tests, those offering routine syphilis tests to all pregnant women and those routinely offering both syphilis and HIV tests.

The study area comprised Mwanza city and Magu district to the East of the city which is bisected by the main road to Kenya and which contains Kisesa ward whose residents participate in the Tazama project's open cohort study [21].

**Figure 1:**
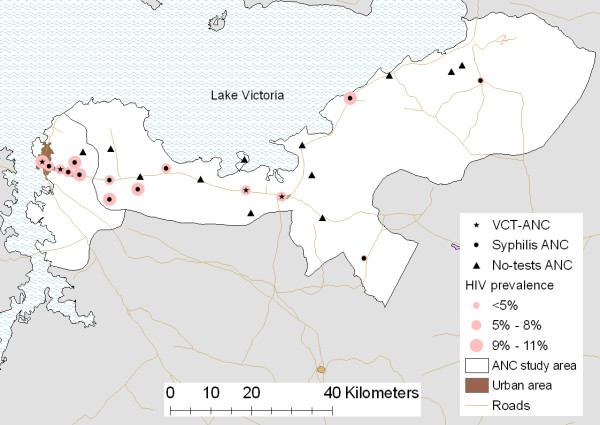
**Location of 2006 study clinics and HIV prevalence at clinics where we collected HIV status**.

In 2006, there were 42 government ANC in Magu district and Mwanza city. The clinic sample was designed to include: all clinics offering VCT in the area (VCT-ANC N = 4) and all clinics offering syphilis tests in the area (Syphilis-ANC N = 11) and 12 of the clinics in the study area that did not offer any diagnostic tests (No-tests-ANC N = 27). The no-test-ANC were selected such that we included all the ANC which women in our study area could easily attend and so we could investigate attendance patterns (Figure 1). We included all the clinics serving the residents of Kisesa ward. All 11 clinics from the previous rounds were included (3 VCT-ANC and 8 Syphilis-ANC).

### 2006 Data collection

Data were collected between March and July 2006 in 27 clinics. The data used in this paper come from the 15 clinics which routinely drew blood during ANC consultations: four which offered VCT and eleven which offered only syphilis tests.

Following national protocol at the time the target sample size was 300 women in each clinic, with every woman attending ANC asked to participate. In the busiest clinic every third woman was asked to participate to maintain a manageable workload for the survey staff.

Women were eligible for the study if they were attending ANC (for a first or subsequent visit) and had not previously been interviewed. In all 15 clinics, behavioural data, HIV status and syphilis status were collected.

Ethical approval was given by the Tanzanian Medical Research Coordinating Committee, WHO and the London School of Hygiene & Tropical Medicine.

Women gave informed consent to be interviewed. In all clinics, women who agreed to participate were asked at the end of the interview whether they would allow us to know the result of their syphilis test. In clinics which also offered VCT, women were asked if they were planning to have an HIV test that day and, if so, whether they would allow us know their HIV status. Women in syphilis only clinics who consented to share their syphilis results with us were also tested anonymously for HIV, in line with national surveillance procedures.

Questionnaire and test data were anonymised on collection in the clinic and later identified and matched using study numbers. HIV tests were conducted on dried blood spots taken from the blood samples provided for syphilis testing.

HIV results were based on the results of two tests (Enzygnost [22] and Uniform 2 [23]) carried out at the National Institute for Medical Research (NIMR); we did not use the results of rapid HIV tests done in the VCT clinics. Syphilis testing was done in the clinics using Rapid Plasma Reagin [24] (RPR) and results were recorded on a special study form.

The questionnaire (see Additional file 1) was administered by regular clinic staff. We aimed to have female interviewers but the extended period of data collection and clinic staffing schedules meant that some interviews (13%) were carried out by male staff when no female staff were available. Participants were asked about their childbearing experience, their marital and cohabitation history, whether they had more than one sexual partner in the last year and whether the father of the baby had more than one sexual partner in the last year, their reasons for choosing the clinic, and prior history of syphilis and HIV testing.

### Geographic data

We obtained co-ordinates for each ANC and village in the study area from a WHO PSAM survey [25] conducted in 2005, a gazetteer from the International Potato Center [26] and from Google Earth [27]. We calculated straight line distance between places of residence and ANC.

### Statistical methods

Data were double-entered in dBase IV. Questionnaire, syphilis and HIV test results were linked by study number. All analysis was done in Stata 10 [28]. Data are clustered at the clinic level so, for analysis of HIV outcomes, standard errors were adjusted for clustering using Stata's survey commands [28].

Bivariate comparisons were conducted using Pearson's Chi-squared test [29]. Multiple logistic regression, adjusted for survey design, was used to investigate the factors associated with HIV infection. Time to event analyses (times to first sex, first marriage and interval between first sex and first marriage) were carried out using cox regression adjusted for the clustering at the clinic level.

Confidence intervals were estimated but should be interpreted with caution since, with the exception of the busiest clinic, these results are not based on a sample survey but on all women attending ANC. The purposive sampling of clinics means that the results are not representative of all women or of all ANC.

Models for the correlates of HIV infection were constructed hierarchically. Separate models were built for demographic, geographical and behavioural factors, using all the characteristics associated with HIV in the bivariate analysis. Factors which remained important in the adjusted models were then combined into one final model.

In the previous rounds, analysis of factors associated with HIV was restricted to women under 25 and we included a latent variable in the logistic regression models for the HIV prevalence in women aged 25 and over seen at the same clinic. We repeated this analysis on the 2006 data.

## Results

Response rates were high, more than 99% of women eligible for each component (interview, syphilis test, and HIV test) consented to participate. Three women refused syphilis tests and 111 women were not eligible because they had already been tested. Three different women refused consent for HIV tests and 142 women were not eligible for HIV tests: 25 had already been tested, 65 were seen in VCT clinics but not planning to take an HIV test and 52 had already had a syphilis test and so there was no clinical reason to draw blood. One woman's HIV result was discarded because no confirmatory test was done.

Complete data were obtained for 3,780 women (93% of eligible women). In eight clinics low attendance rates meant that fewer than 300 women could be recruited in the time available (range from 66 to 274).

### HIV prevalence & trends 2000-2006

Prevalence among 3,839 women tested for HIV in 2006 was 7.6%. HIV prevalence was highest among women seen in urban clinics (8.5%), intermediate in roadside clinics (7.7%) and lowest in remote rural clinics (4.6%). HIV prevalence increased with age from 3% in women aged under 20 to 9.6% in women aged between 25 and 34, but was lower in women aged over 35. A similar trend was seen with parity; prevalence was lowest (5%) among women expecting their first child, increased to 10% among women expecting their third and decreased among women with higher parity. This is consistent with selection effects caused by sub-fertility observed in HIV infected women [30].

**Figure 2:**
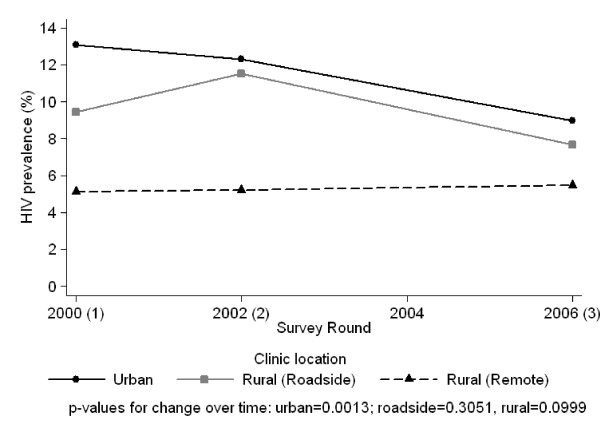
**Trend in HIV prevalence over time by location of clinic (data from the 11 clinics that participated in all rounds)**.

At the 11 clinics involved in every round, HIV prevalence was 7.4%. In urban clinics there had been a steady decline since 2000 (p = 0.0013). In roadside clinics the 2006 estimate was lower than that from 2002 but this could be due to chance (p = 0.3) (Figure 2). There has been no change in the rural clinics, which had a lower prevalence to start with.

Comparison of ANC surveillance results with those from the nationally representative HIV/AIDS Indicator Surveys (AIS 2003/4 [31] & AIS 2007 [32]) and the local community based surveys carried out in Kisesa ward by the Tazama project (Table 1) show that the ANC results are very similar to those obtained for women aged 15-49 in the AIS. The AIS sample was not designed to provide regional estimates for pregnant women or by urban/rural residence, hence the wide confidence intervals in Table 1. Even at the regional level the confidence intervals are wide. Comparison with the Tazama sero-survey results shows that the ANC survey found higher prevalence among pregnant women from Kisesa ward than the community surveys conducted before and shortly after the ANC survey. For men and women aged 15-49 the sero-survey results concur with the AIS estimates. The ANC estimate for all women in 2006 lies between the 2003/4 and 2007 sero-survey estimates.

**Table 1:** HIV prevalence from AIS, ANC surveillance and the Tazama project.

	2003/4 AIS Mwanza region	2007 AIS Mwanza region	2006 ANC all women	2006 ANC Kisesa residents	2003/4 sero survey (4)	2007 sero survey (5)
**Men 15-49**	7.5 (4.7-11.6)	3.4 (2.2-6.1)	-	-	7.4 (6.0-8.8)	4.8 (4.1-5.6)
**Women 15-49**	7 (4.3-11.2)	7.1 (5.2-9.6)	-	-	7.0 (5.9-8.2)	7.8 (7.0-8.6)
**Rural women 15-49**	7.4 (4.3-12.4)	5.3 (3.9-7.1)	-	-	7.0 (5.2-8.8)	8.7 (7.4-10.0)
**Urban women 15-49**	5.9 (1.9-16.9)	14.9 (11.2-19.5)	-	-	7.1 (5.5-8.6)	6.3 (5.3-7.4)
**Pregnant women**	10.31 (4.9-19.5)	4.4 (1.1-16.1)	7.6 (6.3-9.3)	9.1 (6.8-12.1)	5.1 (2.0-8.2)	6.4 (4.0-8.9)

Cells give HIV prevalence and 95% confidence intervals in brackets.

### Geography of HIV and Syphilis infection

**Figure 3:**
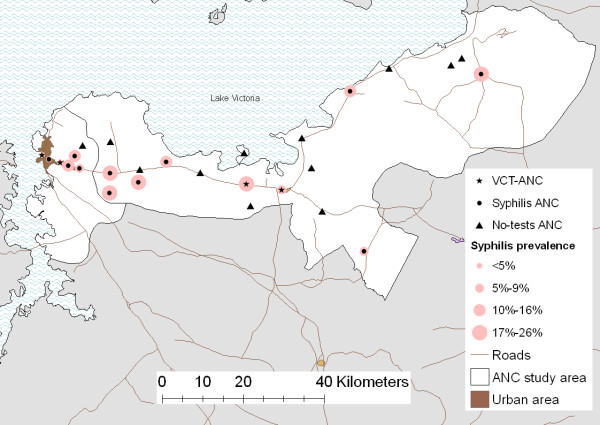
Syphilis prevalence at study clinics in 2006

HIV prevalence tended to be highest in clinics located in Mwanza city and on the main road (Figure 1). Syphilis prevalence was lowest in urban areas and markedly higher elsewhere (Figure 3).

Unlike earlier rounds, neither clinic location nor place of residence were significantly associated with HIV infection. Prevalence was 6.6% for women who lived in remote rural areas compared to 8.4% for urban women and 8.2% for women who lived in rural roadside areas (p-value = 0.279).

Syphilis prevalence was strongly associated with both clinic location and place of residence. Syphilis prevalence was highest among remote rural residents, of whom 16% overall were infected compared to 7% of urban residents and 13% of rural roadside residents (p = 0.007). These differences were attenuated by clinic choice because rural women who attended urban clinics had a prevalence of 12% and roadside women who attended rural clinics had a prevalence of 19%. This may be explained by greater availability and use of diagnostic and treatment services in urban compared to rural areas.

There is no correlation between the prevalence of syphilis and HIV infections. Infection with both syphilis and HIV is correspondingly rare (1.4% of women).

### Changes over time in characteristics of women

We compared characteristics of women attending the 11 clinics participating in all 3 surveillance rounds. The same questionnaire was used in the first two rounds. Although the questionnaire used in 2006 included extra questions on clinic choice, the socio-demographic and sexual behaviour questions were comparable across rounds.

Women attending ANC in the later rounds were older (median age 25 v. 24 in previous rounds). Most fathers were aged between 25 and 34 and the distribution changed little over the rounds.

In 2006, far fewer women reported that they had never been married compared to previous rounds (16%, 19% and 6% in rounds 1,2, and 3 respectively p-value = 0.0021). Women in round 3 appear to have had more education than those in earlier rounds (15% with no education in round 3 compared to 19% in round 1).

Gravidity did not change, but the proportion of women who reported more previous pregnancies than live births, indicative of late miscarriage or stillbirth, decreased from 16% in the first round to 9% in the last (p = 0.005).

The proportion reporting more than one sexual partner in the last year increased from 5% to 8% between the first and second rounds and then decreased to 4% in the third round (p = 0.009).

**Figure 4:**
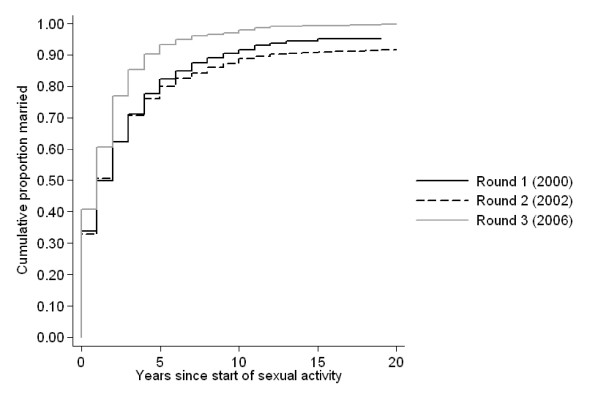
Years spent single and sexually active before marriage by survey round

The number of years spent sexually active before marriage was one of the strongest predictors of HIV status in the first two rounds. Figure 4 shows that women in round 3 spent less time sexually active before marriage compared to women in round 1 (HR from Cox model 1.36, p = 0.001). This is not explained by a change in the median age at first sex, which was 17 in all rounds, but by a fall in the median age at first marriage from 19 in the first two rounds to 18 in the third round (HR from Cox regression 1.4, p < 0.001).

### Factors associated with HIV infection

In the crude analysis, factors associated with HIV infection were: age group, parity, experience of stillbirth, time since last birth, survival of last born child, time spent sexually active between first sex and marriage, cohabitation status, number of marriages, method of transport to clinic, duration of marriage, number of partners and previous HIV test experience (Table 2). Whether the baby's father had other partners (none, other wives, other women, don't know), age gap between partners, recently moving house and previous place of residence were not important (results not shown). The proportion with more than one partner in the last year increases with time spent in pre-marital sexual activity.

**Table 2:** Factors associated with HIV infection among women of all ages.

				CRUDE		ADJUSTED		
	N	% HIV+	OR	95% CI	p	OR	95% CI	p
**Age group**								
**14-19**	803	3.2	0.40	0.26-0.61	0.000	0.44	0.25-0.76	0.006
**20-24**	1140	7.8	1			1		
**25-29**	903	9.8	1.28	0.93-1.75	0.120	1.28	0.80-2.04	0.277
**30-34**	601	9.7	1.26	0.95-1.67	0.095	1.25	0.79-1.96	0.315
**35+**	392	7.7	0.98	0.69-1.39	0.897	1.15	0.53-2.48	0.707
**Number of pregnancies, grouped**								
**First pregnancy**	932	5.0	0.48	0.31-0.72	0.002	1.70	0.87-3.33	0.114
**gravidity 2**	801	8.2	0.80	0.59-1.10	0.154	0.84	0.58-1.22	0.337
**gravidity 3**	637	10.1	1			1		
**gravidity 4**	804	8.7	0.85	0.63-1.16	0.284	0.68	0.49-0.95	0.026
**gravidity 5+**	665	6.6	0.63	0.44-0.92	0.020	0.57	0.32-1.03	0.062
**Ever had a stillbirth/miscarriage**								
**Never had a stillbirth/miscarriage**	3509	7.4	1					
**Had a stillbirth/miscarriage**	329	9.7	1.35	0.95-1.93	0.091			
**Time of most recent birth**^**1**^								
**No previous births/last birth within 2 years**	1713	5.2	1			1		
**Last birth >2 years ago**	2074	9.4	1.89	1.45-2.47	<0.001	1.71	1.08-2.71	0.026
**Survival of last birth child**^**1**^								
**Last child born still alive/no last born child**	3500	6.9	1			1		
**Last child born died**	287	15.3	2.46	1.75-3.45	<0.001	2.09	1.36-3.22	0.003
**Time between first sex and marriage**^**2**^								
**Years between first sex and marriage**	6153		1.1	1.05-1.15	<0.001	1.08	1.04-1.13	0.001
**Cohabitation status**								
**Never cohabited**	206	9.7	1.39	0.80-2.41	0.224			
**Currently married/cohabiting**	3409	7.2	1					
**Married but not living with baby's father**	138	8.7	1.23	0.56-2.72	0.585			
**Been married but not living with baby's father**	86	16.3	2.51	1.19-5.28	0.019			
**Number of marriages**								
**None or one**	3257	6.4	1					
**Been married more than once**	582	14.4	2.49	1.75-3.53	0.000	2.73	1.76-4.23	0.000
**Had other sexual partners in last 12 months**								
**No other partners**	3689	7.4	1					
**Had >1 partner in last year**	126	12.7	1.81	1.11-2.95	0.020			
**DK/refused to say about partners in last year**	24	4.2	0.54	0.05-6.07	0.595			
**Travelled to clinic by**:								
**Foot**	2782	7.9	1					
**Bicycle**	714	5.2	0.63	0.45-0.89	0.013			
**Bus**	204	10.3	1.33	0.74-2.38	0.310			
**Car/motorbike/canoe**	139	8.6	1.09	0.55-2.17	0.780			
**Ever been tested for HIV**								
**Never previously tested**	3683	7.4	1					
**Tested before this pregnancy**	151	9.9	1.38	0.80-2.37	0.226			
**Tested this pregnancy**	5	60	18.74	4.03-87.2	0.001			
**Married less than a year**								
**Married >1 year**	3182	8.0	1	0.29-0.79				
**Married <1 year**	431	3.9	0.48	0.69-2.05	0.007	0.54	0.32-0.91	0.025
**Never married**	226	9.3	1.19		0.516	1.37	0.73-2.59	0.306
**Personal preference**								
**Chose clinic for other reasons**	3759	7.5	1					
**Chose clinic for personal preference**	80	11.3	1.56	0.98-2.49	0.059			
**Straight line distance to clinic**								
**Distance (km)**	7238		0.97	0.94-0.99	0.023			
***Number of women***			*3839*			*3114*		

In the adjusted model (Table 2) method of transport, experience of stillbirth, duration of marriage, number of partners and previous HIV test experience all ceased to be important predictors of HIV status.

The youngest women were least likely to be infected. Women experiencing their fourth or higher order pregnancy were less likely to be infected. Time since last birth remained strongly associated with infection. Women who had not had a birth in the last two years had twice the odds of being HIV positive compared to women whose last birth was more recent or who had never given birth. Child death was also associated with infection and women whose last child had died were twice as likely to be infected. Two factors which may describe risk of HIV acquisition are the number of marriages and the time spent single and sexually active before the first marriage. The odds of HIV infection are doubled in women who have had two or more marriages compared to women who have had one or none. The odds of infection increase by 10% for each extra year spent sexually active before marriage. This suggests that pre-marital sexual activity is associated with higher risk of being HIV positive but also indicates that the break-up of marriage followed by re-marriage is associated with infection. Recent marriage (within a year of the survey) was protective.

Analysis restricted to women aged under 25 produced very similar results (not shown) but the smaller sample size meant that fewer significant associations were detected. The changing epidemiology of infection, with less difference between rural and urban areas, meant that in the 2006 data it was no longer important to control for the HIV prevalence at the clinic[16]. Therefore the theoretical framework for the model applied to the 2000 and 2002 data is no longer appropriate.

## Discussion

### HIV prevalence and trends

HIV prevalence has declined since 2002 in urban clinics but remained stable in the other clinics. Consequently, the difference in prevalence between urban and rural areas has diminished. Although the geography of HIV infection appears to be changing, the patterns of syphilis infection are more stable and rural areas are still worst affected.

Data from the Kisesa community cohort in Magu district confirm the diminishing difference between urban and rural areas and show that incidence is increasing in the rural parts of Kisesa ward whilst decreasing elsewhere [33].

Comparison with other estimates showed that ANC data provide robust estimates of prevalence among women in the population in this area and highlighted that the AIS is inadequate for understanding regional prevalence trends. Estimated prevalence among pregnant women from Kisesa ward was higher in the ANC survey than the community sero-surveys. This may be because women at ANC were different to those who participated in the sero-survey; comparison of key characteristics showed that women from Kisesa ward and interviewed in ANC were younger, more educated and more likely to be primagravidae than women interviewed in the 2007 sero survey. These differences would be more likely to lower ANC prevalence relative to the sero-survey, the opposite of what is observed. Another explanation for the difference is that none of the ANC serving Kisesa women offered VCT so all HIV testing was anonymous in 2006. One of the four clinics that served Kisesa residents did not offer syphilis testing and therefore we have no HIV data for 25% of the Kisesa residents surveyed in ANCs. If the women who were not tested were predominantly negative, the prevalence would be similar to that estimated in the sero-surveys. The changes in the epidemiology of HIV infection in this region may have minimised differences between the ANC and community based data that arise from geographical variations in prevalence. Other biases may be less important in this setting. Fertility is high in Mwanza region and recent increases in age at first sex mean there is now less variation in the age of sexual debut.

### Characteristics of ANC clients

Some of the changes over time in the socio-demographic characteristics of ANC clients, such as increased level of education, probably reflect changes in the wider population. Other differences, such as later childbearing and earlier age at first marriage, may be indicative of behaviour change in response to the HIV epidemic.

There is some evidence for a decline between 2000 and 2006 in two measures of risky sexual behaviour: the number of women reporting more than one partner in the last year initially increased and then decreased and the amount of time spent single and sexually active has decreased. The change in the reported number of partners is unlikely to have been affected by changes in the questionnaire design since this question was the same in all rounds and asked towards the end of the interview. The change in time spent sexually active between first sex and first marriage could have been partly caused by changes in propensity to report an early age at first sex (or a late age at first marriage) but looking at the ages reported by successive birth cohorts in different survey rounds there is no trend suggestive of changes in reporting bias. This change could have been an artefact of improvements in data collection if more complete information about the timing of events resulted in data becoming available for more low risk women. However no such effect was apparent: age at first sex was missing for 18% of women in the first round, 6% in the second and 13% in the most recent round. Age at first marriage was more complete but followed a similar pattern (10%, 2% and 9%) in the three rounds. Age at first sex, the more sensitive measure, was stable over the three rounds despite differences in the completeness of the data.

Women seen in ANC in 2006 have grown up surrounded by HIV prevention messages, and it seems likely that these changes in behaviour are real. Age at first sex is thought to be increasing for women in the community, but this is not yet reflected in this ANC population that, by definition, excludes virgins.

### Factors associated with HIV infection

Many of the factors associated with HIV infection reflect the dynamic of the epidemic: average age at infection dictates the association with age among women in the clinic; HIV-related infertility is apparent as a protective effect of high parity and a risk for a long preceding birth to pregnancy interval; increased mortality among the children of HIV positive women is evident in the association between child death and HIV infection.

Time since the last birth might also be indicative of exposure to infection if correlated with marital disruption. The association is not affected by the duration of marriage, which suggests HIV related infertility is a more likely explanation.

These data confirm most of our earlier findings. However, two aspects of behaviour, time sexually active before marriage and remarriage patterns, emerge as strongly associated with infection, and point to the complex dynamic interplay between partnership behaviours and HIV risk. In short, the women least likely to be infected were those who married soon after first sex and who stayed married although the benefit of marriage may be confined to the early years as indicated by the lower odds of infection experienced by women married for less than one year compared to those who had been married for longer. This is in keeping with results of similar ANC surveillance from rural Malawi where being in a stable first marriage appears to be protective against HIV infection for young women [18].

The behaviour of the partner is a critical determinant of HIV risk in a marriage. In our analysis the women's assessment of her partner's fidelity was not predictive of her own HIV status, but half the women were not able to give an answer and we do not know the accuracy of reported assessments. The woman's own report of multiple partners in the last year was also unimportant. The proportion reporting more than one partner in the last year (4%) is comparable with the AIS [34] but we cannot rule out under-reporting.

In the analysis of the data from the two previous surveys we restricted analysis of HIV risk to women aged under 25 because this represented recent infections. In this round, the analysis for the under-25 s yielded very similar results to the analysis for all women. The time spent single and sexually active was the only factor from the earlier analysis to remain important in 2006.

Our study design means that these results are not representative of the ANC population, nor of women in the community. The close correspondence between the HIV estimates from these data and others suggests that the women in this study are typical of those in the area. However it remains to be seen whether the associations between HIV status and behavioural characteristics in this study are also seen in the wider community.

### Limitations

We interviewed, but were unable to ascertain HIV and Syphilis status, for the 111 women who had already been tested for syphilis before the study. Most of these women (95) were interviewed at one of four clinics. It seems likely that these women had been tested for syphilis on a visit prior to the start of the study. That the effect was seen in these four clinics, but not the others, is probably explained by the availability of syphilis test kits and drugs: in many clinics these were not available outside of the survey period and therefore women were unlikely to have been tested for syphilis before surveillance started.

We could not test 65 women for HIV because they were not planning on taking an HIV test that day. The women who were seen in VCT-ANC but not planning to take a test were predominantly from the two clinics which had recently introduced testing and were interviewed at the start of the study period when HIV testing had only been underway for a few weeks. The proportion of women not planning a test dropped markedly at these clinics during the surveillance period.

The socio-demographic characteristics of women for whom HIV or Syphilis results were not available did not differ from those for whom we obtained test results and we are therefore confident that failure to ascertain some test results has not influenced the results.

## Conclusions

The epidemiology of HIV infection in this region has changed between 2002 and 2006. Geographical differences in prevalence have diminished. Women attending ANC in urban locations are now less likely to be infected than in earlier surveys.

Syphilis infection has remained confined to rural areas where a substantial proportion of women are infected.

In this survey round, geographic factors have ceased to be correlates of HIV infection and an increased number of proximate, individual level measures have emerged as predictive of HIV status. There is evidence for some behaviour change but given the cross-sectional study design it is not possible to say if the decline in HIV prevalence is related to the changes in behaviour.

## Competing interests

The authors declare that they have no competing interests.

## Authors' contributions

YK, ES, BZ, RI, JC & MU designed the study; YK, PM, DK collected the data; YK, ES, BZ, MU wrote the paper; ES & RI analysed the data; all authors commented on drafts and approved the final version.

## Pre-publication history

The pre-publication history for this paper can be accessed here:

http://www.biomedcentral.com/1471-2458/10/553/prepub

## Supplementary Material

Additional file 1 **Survey Questionnaire in Swahili**. The questionnaire used in ante-natal clinics which offered testing for both Syphilis and HIV. In clinics which did not offer diagnostic tests the last section was omitted. Click here for file
