# Supplementary material for: Trends in HIV & syphilis prevalence and correlates of HIV infection: results from cross-sectional surveys among women attending ante-natal clinics in Northern Tanzania
Source: BMC Public Health. 2010 Sep 13;10:553. doi: 10.1186/1471-2458-10-553 (PMC2946298; doi:10.1186/1471-2458-10-553)
Supplement: Additional file 1 — Survey Questionnaire in Swahili. The questionnaire used in ante-natal clinics which offered testing for both Syphilis and HIV. In clinics which did not offer diagnostic tests the last section was omitted. [file 1471-2458-10-553-S1.DOC]

| **National Institute for Medical Research, Mwanza Centre**  **Mwanza City and Magu District Health Departments**  **Antenatal Clinic Surveillance 2006** | | | | | | | | | | | | | | |  |
| --- | --- | --- | --- | --- | --- | --- | --- | --- | --- | --- | --- | --- | --- | --- | --- |
| **MAELEZO KWA WAHOJAJI/WASAILI**  MSOMEE MHOJIWA TAARIFA YOTE YA RIDHAA YA KUSHIRIKI KWAKE NA MASWALI YOTE YALIYOKO KWENYE DODOSO KWA UANGALIFU. MAJIBU YOTE YAANDIKWE KWENYE MISTARI ILIYOCHORWA NAMNA HII____________  KAMA KUNA CODE NAMBA, UNATAKIWA KUZUNGUSHIA JIBU ATAKALOTAJA. | | | | | | | | | 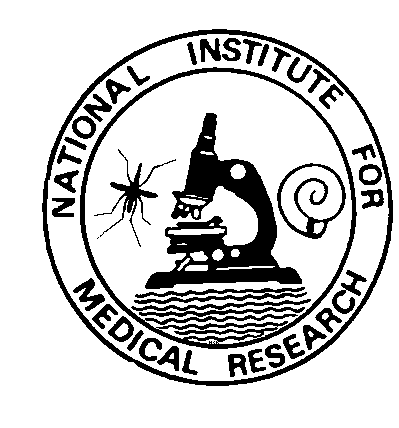 | | | | | |  |
| **Sehemu A:** | | | | | | | | | | | | | | | |
|  | Namba ya Mshiriki/Dodoso: | WEKA NAMBA HAPA | | | | | | | | | | | | | |
|  | Jina la kliniki  (jaza mapema)⋅⋅⋅⋅⋅⋅⋅⋅⋅⋅⋅⋅⋅⋅⋅⋅⋅⋅⋅⋅⋅⋅⋅⋅⋅⋅⋅⋅⋅⋅⋅⋅ | ____________________________ | | | | | | | | | | | | | |
|  | Jina la mhojaji ⋅⋅⋅⋅⋅⋅⋅⋅⋅⋅⋅⋅⋅⋅⋅⋅⋅⋅⋅⋅⋅⋅⋅⋅⋅⋅⋅⋅⋅⋅⋅⋅⋅ | ___________________________ | | | | | | | | | | | | | |
|  | Tarehe ya leo ⋅⋅⋅⋅⋅⋅⋅⋅⋅⋅⋅⋅⋅⋅⋅⋅⋅⋅⋅⋅⋅⋅⋅⋅⋅⋅⋅⋅⋅⋅ | Siku | | ______ | Mwezi | | | ____________ | | | Mwaka | | ___________ | | |
|  | Muda wa kuanza mahojiano⋅⋅ | _________________ | | | | | |  | | |  | |  | | |
| **MSOMEE MHOJIWA MAHOJIANO PAMOJA NA KADI YA RIDHAA YA KUKUBALI KUSHIRIKI KWAKE.** | | | | | | | | | | | | | | | |
|  | Je, Mhojiwa amekubali kushiriki?  **(ZUNGUSHIA JIBU MOJA NA UWEKE SAHIHI)** | | Ndiyo⋅⋅⋅⋅⋅⋅⋅⋅⋅⋅⋅⋅⋅⋅⋅⋅⋅⋅⋅⋅⋅⋅⋅ | | | | 1 ___________ | | | **🡺Sehemu B** | | | | | |
|  |  |  | Hapana⋅⋅⋅⋅⋅⋅⋅⋅⋅⋅⋅⋅⋅⋅⋅⋅⋅⋅⋅ | | | | 2 ____________ | | | **🡺Sehemu E** | | | | | |
| **Sehemu B:** | | | | | | | | | | | | | | | |
| **ANGALIA KADI YA RIDHAA. KAMA JIBU NI “NDIYO” MWULIZE MHOJIWA MASWALI YAFUATAYO:** | | | | | | | | | | | | | | | |
|  | Je, ulizaliwa lini? | | | | | Siku⋅⋅⋅⋅⋅⋅⋅⋅⋅⋅⋅⋅⋅⋅⋅⋅⋅⋅⋅⋅⋅⋅⋅⋅⋅⋅⋅⋅⋅⋅⋅⋅ | | | | | | ____ | |  | |
|  | **(ANDIKA 98 KAMA MHOJIWA HAJUI/HAKUMBUKI SIKU MWEZI NA MWAKA)** | | | | | Mwezi⋅⋅⋅⋅⋅⋅⋅⋅⋅⋅⋅⋅⋅⋅⋅⋅⋅⋅⋅⋅⋅⋅⋅⋅⋅⋅⋅⋅ | | | | | | ____ | |  | |
|  |  |  |  |  |  | Mwaka⋅⋅⋅⋅⋅⋅⋅⋅⋅⋅⋅⋅⋅⋅⋅⋅⋅⋅⋅⋅⋅⋅⋅⋅⋅⋅⋅ | | | | | | ____ | |  | |
|  | Je, una umri wa miaka mingapi kwa sasa?⋅⋅⋅⋅⋅⋅⋅⋅⋅⋅⋅⋅⋅⋅⋅⋅⋅ | | | | | Umri (katika miaka) | | | | | | ____ | |  | |
|  | Je, kwa sasa unaishi kata (Mwanza City kliniki) au kijiji (Magu kliniki) gani?⋅  **(ANDIKA NAMBA YA CODE KUTOKA KWENYE KADI 1. KAMA SEHEMU ANAYOISHI HAIKUORODHESHWA KWENYE KADI 1, ANDIKA JINA LA SEHEMU ANAYOISHI PAMOJA NA WILAYA YAKE)** | | | | | | | | | | | ____ | |  | |
|  | Je, una muda gani unaishi hapo nyumbani? | | | | | Siku zote naishi hapo⋅⋅⋅⋅⋅⋅⋅⋅⋅⋅⋅⋅⋅⋅⋅⋅⋅⋅⋅⋅⋅⋅⋅⋅⋅⋅⋅⋅⋅⋅⋅⋅ | | | | | | 99 | | 🡺 **Q12** | |
|  |  | | | | | Miaka⋅⋅⋅⋅⋅⋅⋅⋅⋅⋅⋅⋅⋅⋅⋅⋅⋅⋅⋅⋅⋅⋅⋅⋅⋅⋅⋅⋅⋅⋅ | | | | | | ____ | |  | |
|  |  | | | | | Miezi⋅⋅⋅⋅⋅⋅⋅⋅⋅⋅⋅⋅⋅⋅⋅⋅⋅⋅⋅⋅⋅⋅⋅⋅⋅⋅⋅⋅⋅⋅⋅⋅ | | | | | | ____ | |  | |
|  |  | | | | | Ninaishi kwa muda | | | | | | 77 | |  | |
|  | Je, ulikuwa unaishi wapi kabla ya hapo?  **(ANDIKA NAMBA YA CODE KUTOKA KWENYE KADI 1. KAMA SEHEMU ALIYOTAJA HAIPO KWENYE CODE ANDIKA JINA LA SEHEMU HIYO PAMOJA NA WILAYA YAKE)**  **_ _ _ _ _ _ _ _ _ _ _ _ _ _ _ _ _ _ _ _** | | | | | | | | | | | _____ | |  | |

|  | | Je, umewahi kupata mimba mara ngapi ikiwa ni pamoja na mimba hii ya sasa? | | Idadi ya mimba⋅⋅⋅⋅⋅⋅⋅⋅⋅⋅⋅⋅⋅⋅ | | | | ____ | | |  | | | |
| --- | --- | --- | --- | --- | --- | --- | --- | --- | --- | --- | --- | --- | --- | --- |
|  | | Kwa ujumla, kabla ya mimba hii umewahi kuzaa mtoto/ watoto hai mara ngapi?  **(ANDIKA 0 KAMA NI MIMBA YA KWANZA)** | | Idadi ya uzazi/ vizazi hai⋅⋅⋅⋅⋅⋅⋅⋅⋅⋅⋅⋅⋅⋅⋅⋅⋅⋅⋅⋅⋅⋅⋅⋅⋅⋅⋅⋅⋅⋅⋅⋅⋅⋅⋅⋅⋅⋅⋅ | | | | ____ | | |  | | | |
| **ANGALIA KAMA AMEWAHI KUZAA. KAMA HAJAWAHI KUZAA, HAKIKISHA NA UENDE | | 🡺Q16** | | | | | | | | | | | | | | |
|  | | Je, mtoto wako wa mwisho ulimzaa lini? | | | | Siku⋅⋅⋅⋅⋅⋅⋅⋅⋅⋅⋅⋅⋅⋅⋅⋅⋅⋅⋅⋅⋅⋅⋅⋅⋅⋅⋅⋅⋅⋅⋅⋅⋅ | _____ | | | | |  | | |
|  | | **( ANDIKA 98 KAMA** | | | | Mwezi⋅⋅⋅⋅⋅⋅⋅⋅⋅⋅⋅⋅⋅⋅⋅⋅⋅⋅⋅⋅⋅⋅⋅⋅⋅⋅⋅⋅⋅ | _____ | | | | |  | | |
|  | | **MHOJIWA HAJUI/HAKUMBUKI** | | | | Mwaka⋅⋅⋅⋅⋅⋅⋅⋅⋅⋅⋅⋅⋅⋅⋅⋅⋅⋅⋅⋅⋅⋅⋅⋅⋅⋅⋅ | _____ | | | | |  | | |
|  | | **SIKU, MWEZI NA MWAKA )** | | | | AU |  | | | | |  | | |
|  | |  | | | | Miezi iliyopita ⋅⋅⋅⋅⋅⋅⋅⋅⋅⋅⋅⋅⋅ | _____ | | | | |  | | |
|  | |  | | | | Miaka iliyopita⋅⋅⋅⋅⋅⋅⋅⋅⋅⋅⋅⋅⋅ | _____ | | | | |  | | |
|  | | Je, mtoto huyu bado yuko hai? | | | | Yuko hai⋅⋅⋅⋅⋅⋅⋅⋅⋅⋅⋅⋅⋅⋅⋅⋅⋅⋅⋅⋅⋅⋅⋅⋅ | 1 | | | | |  | | |
|  | |  | | | | Hayuko hai⋅⋅⋅⋅⋅⋅⋅⋅⋅⋅⋅⋅⋅⋅⋅⋅⋅⋅⋅ | 2 | | | | |  | | |
|  | | Je, hii ni mara yako ya kwanza kuhudhuria kliniki kwa mimba hii? | | | | Ndiyo⋅⋅⋅⋅⋅⋅⋅⋅⋅⋅⋅⋅⋅⋅⋅⋅⋅⋅⋅⋅⋅⋅⋅⋅⋅⋅⋅⋅⋅⋅ | 1 | | | | | **🡺Sehemu C** | | |
|  | |  |  |  |  | Hapana⋅⋅⋅⋅⋅⋅⋅⋅⋅⋅⋅⋅⋅⋅⋅⋅⋅⋅⋅⋅⋅⋅⋅⋅⋅⋅ | 2 | | | | | **🡺Q17** | | |
|  | | Je, umewahi kushiriki katika utafiti huu kwa mimba hii? | | | | Ndiyo⋅⋅⋅⋅⋅⋅⋅⋅⋅⋅⋅⋅⋅⋅⋅⋅⋅⋅⋅⋅⋅⋅⋅⋅⋅⋅⋅⋅⋅⋅ | 1 | | | | | **🡺Sehemu E** | | |
|  | |  |  |  |  | Hapana⋅⋅⋅⋅⋅⋅⋅⋅⋅⋅⋅⋅⋅⋅⋅⋅⋅⋅⋅⋅⋅⋅⋅⋅⋅⋅ | 2 | | | | | **🡺Q18** | | |
|  | | Je, umewahi kuhudhuria kliniki ya wajawazito mara ngapi kwa mimba hii? | | | | | ____ | | | | | **🡺Q19** | | |
|  | | Je, kwa mara ya kwanza ulihudhuria kliniki ipi kwa mimba hii?  **(ANDIKA NAMBA YA CODE KUTOKA KWENYE KADI 2)** | | | | | ____ | | | | | **🡺Sehemu C** | | |
| **Sehemu C:** | | | | | | | | | | | | | | |
| **MWULIZE MHOJIWA MASWALI YAFUATAYO:** | | | | | | | | | | | | | | |
| 1. Q | Je, kwa sehemu kubwa umetumia usafiri gani kwa leo kuja hapa kliniki? | | | | Nimetembea⋅⋅⋅⋅⋅⋅⋅⋅⋅⋅⋅⋅⋅⋅⋅⋅⋅ | | | | | 1 |  | | | |
|  |  |  |  |  | Basi⋅⋅⋅⋅⋅⋅⋅⋅⋅⋅⋅⋅⋅⋅⋅⋅⋅⋅⋅⋅⋅⋅⋅⋅⋅⋅⋅⋅⋅⋅⋅⋅⋅⋅ | | | | | 2 |  | | | |
|  | **(ZUNGUSHIA JIBU MOJA)** | | | | Baiskeli⋅⋅⋅⋅⋅⋅⋅⋅⋅⋅⋅⋅⋅⋅⋅⋅⋅⋅⋅⋅⋅⋅⋅⋅⋅⋅⋅ | | | | | 3 |  | | | |
|  |  | | | | Gari ndogo ⋅⋅⋅⋅⋅⋅⋅⋅⋅⋅⋅⋅⋅⋅⋅⋅⋅⋅⋅ | | | | | 4 |  | | | |
|  |  | | | | Mtumbwi⋅⋅⋅⋅⋅⋅⋅⋅⋅⋅⋅⋅⋅⋅⋅⋅⋅⋅⋅⋅⋅⋅⋅⋅ | | | | | 5 |  | | | |
|  |  | | | | Pikipiki⋅⋅⋅⋅⋅⋅⋅⋅⋅⋅⋅⋅⋅⋅⋅⋅⋅⋅⋅⋅⋅⋅⋅⋅ | | | | | 6 |  | | | |
| 1. Q | Je, ni kwa nini umekuja kupata huduma katika  kliniki hii na haukwenda sehemu nyingine? | | | | Hapa ni karibu zaidi⋅⋅⋅ | | | | | A |  | | | |
|  |  |  |  |  | Ni rahisi kufika hapa⋅ | | | | | B |  | | | |
|  |  |  |  |  | Ni kliniki nzuri sana⋅⋅⋅ | | | | | C |  | | | |
|  | **(ZUNGUSHIA MAJIBU YOTE ATAKAYOKUTAJIA)** | | | | Kuna matibabu ya  magonjwa ya ngono⋅⋅ | | | | | D |  | | | |
|  |  | | | | Kliniki hutoa huduma ya PMTCT⋅⋅⋅⋅⋅⋅⋅⋅⋅⋅⋅⋅⋅⋅⋅⋅⋅⋅⋅⋅⋅⋅ | | | | | E |  | | | |
|  | **_ _ _ _ _ _ _ _ _ _ _ _ _ _ _ _ _ _ _ _ _** | | | | Nyingine(Taja)⋅⋅⋅⋅⋅⋅⋅⋅⋅⋅⋅⋅⋅ | | | | | F |  | | | |
| 1. Q | Je, ni kiwango gani cha juu cha elimu ulichofikia?  **(ANDIKA DARASA ALILOFIKIA NA ZUNGUSHIA CODE HUSIKA. USIANDIKE NAMBA ZA KIRUMI KWENYE DARASA NA KIDATO. )** | | | | Sikusoma kabisa⋅⋅⋅⋅⋅⋅⋅⋅⋅ | | | | | 0 | | |  | |
|  |  |  |  |  | Darasa⋅⋅⋅⋅⋅⋅⋅⋅⋅⋅⋅⋅⋅⋅⋅⋅⋅⋅⋅⋅⋅⋅⋅⋅⋅⋅⋅⋅ | | | | | _____ | | |  | |
|  |  |  |  |  | Kidato⋅⋅⋅⋅⋅⋅⋅⋅⋅⋅⋅⋅⋅⋅⋅⋅⋅⋅⋅⋅⋅⋅⋅⋅⋅⋅⋅⋅⋅⋅ | | | | | _____ | | |  | |
|  | **_ _ _ _ _ _ _ _ _ _ _ _ _ _ _ _ _ _ _ _ _** | | | | Nyingine(Taja)⋅⋅⋅⋅⋅⋅⋅⋅⋅⋅⋅⋅⋅ | | | | | 88 | | |  | |
| **MSOMEE MHOJIWA MAELEZO YAFUATAYO:**  Kwa sasa napenda tuzungumzie kuhusu mimba hii uliyonayo pamoja na baba wa mtoto aliyemo tumboni | | | | | | | | | | | | | | |
|  | | Je, mwanamme huyu ana umri wa miaka mingapi?  **(KAMA MHOJIWA HAJUI UMRI MWAMBIE AKISIE)** | Umri katika Miaka⋅⋅⋅⋅⋅⋅⋅⋅⋅⋅⋅ | | | | | | _____ | | | | |  |
|  | |  | AU  Mwaka⋅⋅⋅⋅⋅⋅⋅⋅⋅⋅⋅⋅⋅⋅⋅⋅⋅⋅⋅⋅⋅⋅⋅⋅⋅⋅⋅⋅⋅⋅⋅⋅⋅ | | | | | | _____ | | | | |  |
|  | |  | AU  Ni mdogo kwa miaka⋅⋅⋅⋅⋅ | | | | | | _____ | | | | |  |
|  | |  | AU  Ni mkubwa kwa miaka⋅ | | | | | | _____ | | | | |  |
|  | |  | Sijui/Sikumbuki⋅⋅⋅⋅⋅⋅⋅⋅⋅⋅⋅⋅⋅⋅⋅ | | | | | | 99 | | | | |  |
|  | | Je, anaishi wapi? | Ninaishi naye⋅⋅⋅⋅⋅⋅⋅⋅⋅⋅⋅⋅⋅⋅⋅⋅⋅⋅⋅⋅ | | | | | | 1 | | | | |  |
|  | | **(KAMA MHOJIWA ATATAJA JINA LA SEHEMU ANAYOISHI, HAKIKISHA KAMA WANAISHI PAMOJA)** | Tunaishi kijiji/sehemu moja⋅⋅⋅⋅⋅⋅⋅⋅⋅⋅⋅⋅⋅⋅⋅⋅⋅⋅⋅⋅⋅⋅⋅⋅⋅⋅⋅⋅⋅⋅⋅⋅⋅⋅⋅⋅⋅ | | | | | | 2 | | | | |  |
|  | |  | Anaishi sehemu nyingine⋅⋅⋅⋅⋅⋅⋅⋅⋅⋅⋅⋅⋅⋅⋅⋅⋅⋅⋅⋅⋅⋅⋅⋅⋅⋅⋅⋅⋅⋅ | | | | | | 3 | | | | |  |
|  | | Je, umeolewa na mwanamme huyo? | Ndiyo⋅⋅⋅⋅⋅⋅⋅⋅⋅⋅⋅⋅⋅⋅⋅⋅⋅⋅⋅⋅⋅⋅⋅⋅⋅⋅⋅⋅⋅⋅⋅⋅⋅⋅⋅⋅ | | | | | | 1 | | | | | 🡺**Q27** |
|  | |  | Hapana⋅⋅⋅⋅⋅⋅⋅⋅⋅⋅⋅⋅⋅⋅⋅⋅⋅⋅⋅⋅⋅⋅⋅⋅⋅⋅⋅⋅⋅⋅⋅⋅ | | | | | | 2 | | | | |  |
|  | | Je, umewahi kuolewa? | Ndiyo⋅⋅⋅⋅⋅⋅⋅⋅⋅⋅⋅⋅⋅⋅⋅⋅⋅⋅⋅⋅⋅⋅⋅⋅⋅⋅⋅⋅⋅⋅⋅⋅⋅⋅⋅⋅ | | | | | | 1 | | | | |  |
|  | |  | Hapana⋅⋅⋅⋅⋅⋅⋅⋅⋅⋅⋅⋅⋅⋅⋅⋅⋅⋅⋅⋅⋅⋅⋅⋅⋅⋅⋅⋅⋅⋅⋅ | | | | | | 2 | | | | | 🡺**Q30** |
|  | | Je, ulikuwa na miaka mingapi wakati unaolewa na mme huyu uliyenaye sasa/ulipoolewa kwa mara ya mwisho?  **(ANDIKA 98 KAMA HAJUI)** | Umri (katika miaka)⋅⋅⋅⋅⋅⋅⋅ | | | | | | _____ | | | | |  |
|  | |  | AU  Mwaka⋅⋅⋅⋅⋅⋅⋅⋅⋅⋅⋅⋅⋅⋅⋅⋅⋅⋅⋅⋅⋅⋅⋅⋅⋅⋅⋅⋅⋅⋅⋅⋅⋅ | | | | | | _____ | | | | |  |
|  | |  | AU  miaka mingapi iliyopita | | | | | | _____ | | | | |  |
|  | |  | Sijui/Sikumbuki⋅⋅⋅⋅⋅⋅⋅⋅⋅⋅⋅⋅⋅⋅⋅ | | | | | | 99 | | | | |  |
|  | | Je, umewahi kuolewa mara ngapi? | Idadi ya ndoa :⋅⋅⋅⋅⋅⋅⋅⋅⋅⋅⋅⋅⋅⋅⋅⋅ | | | | | | _____ | | | | | **Kama ni mara 1 🡺Q30** |
|  | | Je, ulikuwa na miaka mingapi wakati ulipoolewa kwa mara ya kwanza? | Umri (katika miaka)⋅⋅⋅⋅⋅⋅⋅ | | | | | | _____ | | | | |  |
|  | |  | AU  miaka mingapi iliyopita | | | | | | _____ | | | | |  |
|  | |  | AU  Mwaka⋅⋅⋅⋅⋅⋅⋅⋅⋅⋅⋅⋅⋅⋅⋅⋅⋅⋅⋅⋅⋅⋅⋅⋅⋅⋅⋅⋅⋅⋅⋅⋅⋅ | | | | | | _____ | | | | |  |
|  | |  | Sijui/Sikumbuki⋅⋅⋅⋅⋅⋅⋅⋅⋅⋅⋅⋅⋅⋅⋅ | | | | | | 99 | | | | |  |

|  | Ulikuwa na umri wa miaka mingapi ulipofanya mapenzi kwa mara ya kwanza? | Umri kwa miaka⋅⋅⋅⋅⋅⋅⋅⋅⋅⋅⋅⋅⋅⋅ | _____ | |  |
| --- | --- | --- | --- | --- | --- |
|  |  | AU  Mwaka⋅⋅⋅⋅⋅⋅⋅⋅⋅⋅⋅⋅⋅⋅⋅⋅⋅⋅⋅⋅⋅⋅⋅⋅⋅⋅⋅⋅⋅⋅⋅⋅⋅ | _____ | |  |
|  |  | Wakati naolewa mara ya kwanza⋅⋅⋅⋅⋅⋅⋅⋅⋅⋅⋅⋅⋅⋅⋅⋅⋅⋅⋅⋅⋅⋅⋅⋅⋅⋅ | 96 | |  |
|  |  | Sijui/Sikumbuki⋅⋅⋅⋅⋅⋅⋅⋅⋅⋅⋅⋅⋅⋅⋅ | 99 | |  |
|  | Je, Katika miezi 12 iliyopita, unadhani baba wa mtoto amefanya mapenzi na mwanamke mwingine zaidi yako? | Ndiyo, mke mwingine⋅⋅⋅⋅ | 1 | |  |
|  |  | Ndiyo, wanawake wengine⋅⋅⋅⋅⋅⋅⋅⋅⋅⋅⋅⋅⋅⋅⋅⋅⋅⋅⋅⋅⋅⋅⋅⋅⋅⋅⋅⋅⋅⋅ | 2 | |  |
|  |  | Hapana⋅⋅⋅⋅⋅⋅⋅⋅⋅⋅⋅⋅⋅⋅⋅⋅⋅⋅⋅⋅⋅⋅⋅⋅⋅⋅⋅⋅⋅⋅⋅⋅ | 3 | |  |
|  |  | Sijui ⋅⋅⋅⋅⋅⋅⋅⋅⋅⋅⋅⋅⋅⋅⋅⋅⋅⋅⋅⋅⋅⋅⋅⋅⋅⋅⋅⋅⋅⋅⋅⋅⋅⋅⋅⋅⋅ | 99 | |  |
|  | Je, katika miezi 12 iliyopita umefanya mapenzi na mtu mwingine yeyote zaidi ya mme wako? | Ndiyo⋅⋅⋅⋅⋅⋅⋅⋅⋅⋅⋅⋅⋅⋅⋅⋅⋅⋅⋅⋅⋅⋅⋅⋅⋅⋅⋅⋅⋅⋅⋅⋅⋅⋅⋅⋅ | 1 | |  |
|  |  | Hapana⋅⋅⋅⋅⋅⋅⋅⋅⋅⋅⋅⋅⋅⋅⋅⋅⋅⋅⋅⋅⋅⋅⋅⋅⋅⋅⋅⋅⋅⋅⋅⋅ | 2 | |  |
|  |  | Sijui/Sina jibu⋅⋅⋅⋅⋅⋅⋅⋅⋅⋅⋅⋅⋅⋅⋅⋅⋅ | 99 | |  |
| **MSOMEE MHOJIWA MAELEZO YAFUATAYO:**  **MASWALI YAFUATAYO YANAHUSU UPIMAJI WA MAAMBUKIZI YA KASWENDE NA VIRUSI VYA UKIMWI(VVU). TUNAPENDA KUFAHAMU TU KAMA UMEWAHI KUPIMA VIPIMO HIVYO NA HATUPENDI KUFAHAMU MATOKEO/AU UNIAMBIE MATOKEO YA KIPIMO/VIPIMO HIVYO.** | | | | | |
|  | Je, umewahi kupima maambukizi ya vimelea vinavyosababisha ugonjwa wa Kaswende? | Ndiyo⋅⋅⋅⋅⋅⋅⋅⋅⋅⋅⋅⋅⋅⋅⋅⋅⋅⋅⋅⋅⋅⋅⋅⋅⋅⋅⋅⋅⋅⋅⋅⋅⋅⋅⋅⋅ | 1 |  | |
|  |  | Hapana⋅⋅⋅⋅⋅⋅⋅⋅⋅⋅⋅⋅⋅⋅⋅⋅⋅⋅⋅⋅⋅⋅⋅⋅⋅⋅⋅⋅⋅⋅⋅⋅ | 2 | 🡺**Q35** | |
|  |  | Sijui/Sikumbuki⋅⋅⋅⋅⋅⋅⋅⋅⋅⋅⋅⋅⋅⋅⋅ | 99 | 🡺**Q35** | |
|  | Je, umewahi kupima maambukizi ya vimelea vinavyosababisha ugonjwa wa Kaswende kwa mimba hii? | Ndiyo⋅⋅⋅⋅⋅⋅⋅⋅⋅⋅⋅⋅⋅⋅⋅⋅⋅⋅⋅⋅⋅⋅⋅⋅⋅⋅⋅⋅⋅⋅⋅⋅⋅⋅⋅⋅ | 1 | **🡺Sehemu E** | |
|  |  | Hapana⋅⋅⋅⋅⋅⋅⋅⋅⋅⋅⋅⋅⋅⋅⋅⋅⋅⋅⋅⋅⋅⋅⋅⋅⋅⋅⋅⋅⋅⋅⋅⋅ | 2 |  | |
|  |  | Sijui/Sikumbuki⋅⋅⋅⋅⋅⋅⋅⋅⋅⋅⋅⋅⋅⋅⋅ | 99 |  | |
|  | Je, umewahi kupima maambukizi ya virusi vya UKIMWI? | Ndiyo⋅⋅⋅⋅⋅⋅⋅⋅⋅⋅⋅⋅⋅⋅⋅⋅⋅⋅⋅⋅⋅⋅⋅⋅⋅⋅⋅⋅⋅⋅⋅⋅⋅⋅⋅⋅ | 1 |  | |
|  |  | Hapana⋅⋅⋅⋅⋅⋅⋅⋅⋅⋅⋅⋅⋅⋅⋅⋅⋅⋅⋅⋅⋅⋅⋅⋅⋅⋅⋅⋅⋅⋅⋅⋅ | 2 | 🡺**Q37** | |
|  |  | Sijui/Sikumbuki⋅⋅⋅⋅⋅⋅⋅⋅⋅⋅⋅⋅⋅⋅⋅⋅ | 99 | 🡺**Q37** | |
|  | Je, umewahi kupima maambukizi ya virusi vya UKIMWI kwa mimba hii? | Ndiyo⋅⋅⋅⋅⋅⋅⋅⋅⋅⋅⋅⋅⋅⋅⋅⋅⋅⋅⋅⋅⋅⋅⋅⋅⋅⋅⋅⋅⋅⋅⋅⋅⋅⋅⋅⋅ | 1 |  | |
|  |  | Hapana⋅⋅⋅⋅⋅⋅⋅⋅⋅⋅⋅⋅⋅⋅⋅⋅⋅⋅⋅⋅⋅⋅⋅⋅⋅⋅⋅⋅⋅⋅⋅⋅ | 2 |  | |
|  |  | Sijui/Sikumbuki⋅⋅⋅⋅⋅⋅⋅⋅⋅⋅⋅⋅⋅⋅⋅⋅ | 99 |  | |

| **Sehemu D:** | | | | | | | | |  |
| --- | --- | --- | --- | --- | --- | --- | --- | --- | --- |
| **SASA NINGEPENDA NIKUULIZE MASWALI, LAKINI NASISITIZA TENA KWAMBA JINA LAKO HALITATUMIKA POPOTE. NIA YA SHUGHULI HII SIYO KUJUA NI NANI ANA TATIZO GANI BALI NI KUJUA TU KIWANGO CHA MATATIZO YA KIAFYA KWA AJILI YA KUBORESHA HUDUMA KWA AKINAMAMA WAJAWAZITO NA JAMII KWA UJUMLA.** | | | | | | | | |  |
|  | Je, utaturuhusu kuandika matokeo ya kipimo chako cha kaswende mwishoni mwa utafiti huu? | | Ndiyo⋅⋅⋅⋅⋅⋅⋅⋅⋅ | 1 | | Weka sticker 1 yenye namba kwenye test tube na nyingine 1 kwenye fomu ya matokeo ya Kaswende | | |  |
|  |  |  | Hapana⋅⋅⋅⋅⋅ | 2 | | Ambatanisha sticker 4 zenye namba mwishoni mwa dodoso na bandika sehemu E kwenye dodoso **🡺Sehemu E** | | |  |
|  | Je, una mpango wa kupima maambukizi ya VVU kwa hiari yako katika kliniki hii? | | Ndiyo⋅⋅⋅⋅⋅⋅⋅⋅ | 1 | |  | | |  |
|  |  |  | Hapana⋅⋅⋅⋅⋅ | 2 | | Ambatanisha sticker 2 nyingine zenye namba mwishoni mwa dodoso na bandika sehemu E kwenye dodoso **🡺Sehemu E** | | |  |
|  | Je, utaturuhusu kuandika matokeo ya kipimo chako cha maambukizi ya VVU mwishoni mwa utafiti huu? | | Ndiyo⋅⋅⋅⋅⋅⋅⋅⋅⋅ | 1 | | Mpatie mshiriki sticker 2 zenye namba ampatie mwuuguzi/mganga anayechukua damu.  Sticker 1 weka kwenye filter paper  na sticker 1 nyingine weka kwenye fomu ya matokeo ya virusi vya UKIMWI (VVU) | | |  |
|  |  |  | Hapana⋅⋅⋅⋅⋅ | 2 | | Ambatanisha sticker 2 nyingine zenye namba mwishoni mwa dodoso na bandika sehemu E kwenye dodoso | | |  |
| **Sehemu E:** | | | | | | | | |  |
|  | | Je, katika mahojiano haya ni lugha gani zaidi iliyotumika? | | | Kiswahili …………… | | 1 |  |  |
|  |  |  |  |  | Kisukuma …………. | | 2 |  |  |
|  | | _ _ _ _ _ _ _ _ _ _ _ _ _ _ _ _ _ | | | Nyingine (Taja)…. | | 3 |  |  |
| **BANDIKA STICKERS AMBAYO HAIKUTUMIKA HAPA** | | | | | | | | | |

**MSHUKURU MAMA KWA KUTOA MUDA WAKE KUONGEA NAWE.**

**MWISHO: MPATIE MAMA KARATASI YA TAARIFA YA MAHOJIANO.**
